# Altered HDL particle in sickle cell disease: decreased cholesterol content is associated with hemolysis, whereas decreased Apolipoprotein A1 is linked to inflammation

**DOI:** 10.1186/s12944-019-1174-5

**Published:** 2019-12-20

**Authors:** Ahmet Yalcinkaya, Selma Unal, Yesim Oztas

**Affiliations:** 1 Department of Medical Biochemistry, Faculty of Medicine, Hacettepe University, Ankara, Turkey; 2 Department of Pediatric Hematology, Faculty of Medicine, Mersin University, Mersin, Turkey

**Keywords:** Sickle cell disease, Hypocholesterolemia, Anemia, Inflammation, Oxidative stress

## Abstract

**Background:**

Hypocholesterolemia is the most frequently encountered lipid abnormality in sickle cell disease (SCD). We enrolled pediatric patients to determine the relationships between lipid profile and parameters of hemolysis, oxidative stress and chronic inflammation in SCD.

**Methods:**

The study involved 35 pediatric SCD patients and 19 healthy controls. Patients were crisis-free and had not received transfusions for the last 3 months. Total cholesterol, triglyceride, HDL-C, LDL-C, VLDL-C, apolipoprotein A1, apolipoprotein B, LCAT, LDH, bilirubin, haptoglobin, iron, ferritin, hemin, serum amyloid A (SAA), myeloperoxidase (MPO), uric acid, ALT and GGT levels were evaluated in patients’ blood.

**Results:**

Patients had hypocholesterolemia depicted by lower levels of total cholesterol, HDL-C, LDL-C, as well as Apolipoprotein A1 and Apolipoprotein B compared to controls. The chronic hemolysis of SCD was evident in patients by higher LDH and bilirubin and almost undetectable haptoglobin levels. Hemin levels (as a measure of oxidized heme) were significantly increased in patients with SCD. Inflammation markers, SAA and MPO, were significantly increased in the patients as well. There were negative correlations between HDL-C and LDH, and Apo A1 and SAA. Hemin was positively correlated to MPO.

**Conclusion:**

Hemolysis was associated with decreased HDL –C, and Inflammation was linked to decreased apolipoprotein A1 levels in our SCD patients. Therefore, we suggest that the HDL particle is altered during the course of the disease. The altered HDL in SCD may become dysfunctional and result with a slowing down of the reverse cholesterol transport.

## Introduction

Sickle cell disease (SCD) is an inherited hemoglobinopathy, caused by a point mutation in the beta globin gene that induces abnormal hemoglobin production (Hemoglobin S, HbS) [1]. The mutant HbS monomers polymerize under hypoxia, acidosis and dehydration, and the erythrocyte gains a sickled shape. Sickle erythrocytes become prone to hemolysis, and patients with SCD present with anemia. Sickle erythrocytes have increased adhesion to each other and vascular endothelium, resulting with vaso-occlusions. Vaso-occlusions lead to ischemia-reperfusion injury and inflammation. Besides acute clinical conditions (painful episodes, crises), severe systemic complications, such as stroke and acute chest syndrome, have been observed in SCD patients, leading to significant morbidity and mortality [2, 3].

SCD is a chronic condition and patients have increased hemolytic, oxidant and inflammatory stress, even at the steady state of the disease [4]. Anemia is a result of chronic hemolysis, induced by continuous sickling of the erythrocyte. This may also induce Hb denaturation and lead to increased oxidative stress through various mechanisms. Of these mechanisms, higher methemoglobin levels, heme and iron release into the circulation, and hemin formation from free heme are the most prominent. Ischemia-reperfusion injury, consumption of endothelial nitric oxide by plasma free hemoglobin and generation of free radicals by heme, hemin and free iron are all involved in the generation of a systemic inflammatory response [4]. Clinically, almost all patients with SCD suffer from various complications at very early ages, including acute chest syndrome, painful crises, cerebrovascular events and endocrine and renal problems.

Hypocholesterolemia is the most frequently encountered lipid abnormality in SCD, with decreases in all lipoprotein cholesterol fractions except VLDL [5, 6]. It occurs as a result of consumption of plasma cholesterol pool, in response to increased erythropoietic activity due to chronic anemia of various etiologies [7]. Erythrocyte lipid peroxidation product, malonyl dialdehyde, was reported to be inversely associated with plasma cholesterol levels by our group [8]. Besides increased oxidative stress and free radical production in SCD may end up with increased oxidation of plasma cholesterol producing oxysterols, such as 7-ketocholesterol. We found increased 7-ketocholesterol levels in the plasma of SCD patients compared to healthy controls [9, 10].

Lipoprotein particles undergo continuous remodeling in the plasma as they have a dynamic equilibrium [11]. Low density lipoprotein cholesterol (LDL-C) and high density lipoprotein cholesterol (HDL-C) levels were reported to decrease in SCD, in addition to some alterations in their composition [12]. Oxidative damage to the components of these lipoprotein particles may also lead to the increased removal of these particles from the circulation by the macrophages [13]. Lipid peroxidation products may affect the function of lipoprotein particles, such as decreased cholesterol uptake by HDL particle (reverse cholesterol transport) and increased plasma elimination of LDL [14].

During acute hemolysis, membrane remnants, particularly cholesterol, are carried to the spleen by the reticuloendothelial system. An acute phase protein, serum amyloid A (SAA) was known to contribute to this process [15]. There is one study that suggested increased SAA levels as an acute phase marker in SCD [16]. However, SAA was not investigated in SCD in line with its mentioned effects on lipid metabolism.

We hypothesized that hypocholesterolemia is associated with chronic hemolysis, oxidative stress and inflammation in the course of SCD. We enrolled pediatric SCD patients (i) to determine the lipid profile and parameters of hemolysis, oxidative stress and chronic inflammation and (ii) to evaluate the relationships between lipid profile and all of these parameters in SCD.

## Material and methods

### Study group

The study group involved pediatric patients aged between 5 and 19 years. The diagnoses of all patients with SCD had been confirmed via hemoglobin electrophoresis, high performance liquid chromatography and mutation analyses. Inclusion criteria were: attending regular follow-up at the pediatric hematology clinic of Mersin University Hospital, having a diagnosis of homozygous SCD (HbSS) or sickle beta thalassemia (HbSß^+^), being crisis-free for at least 3 months, and not having received transfusion(s) during the last 3 months. Exclusion criteria were: suffering from any other chronic disease, having an active infection or an inflammatory condition, not accepting to participate in the study or withdrawing at any point. All patients had been receiving treatment with hydroxyurea.

The control group was comprised of age- and sex-matched healthy children who had applied to the hospital for routine pediatric check-up. Those who declined to participate, those with any acute or chronic illnesses and those using any medications were excluded from the study.

The following clinical characteristics (during the year before their blood withdrawal) of all patients were obtained from patient records: painful episode (crisis), acute chest syndrome, cerebrovascular event, transfusion, and renal, endocrine and cardiac complications. Patients were categorized into 3 groups, according to the number of painful episodes (none, 1–5 times, and > 5 times). The other characteristics were evaluated on a present/absent basis. The presence or absence of renal, endocrine and cardiac complications was defined as a new development of complication or significant worsening of patients’ ongoing problems as reported by a pediatric hematologist (S.U). Acute chest syndrome was defined with the emergence of radiological findings in the presence of respiratory and/or systemic symptoms related to an infection (cough, fever, shortness of breath). Cerebrovascular events were defined according to magnetic resonance imaging that was ordered in the event of clinical suspicion. Renal, endocrine and cardiac complications were defined according their respective imaging/clinical findings (if applicable) and laboratory results (creatinine levels, thyroid/liver/adrenal test results and troponin levels, respectively).

Ethical approval was obtained from Mersin University Clinical Research Ethical Committee (2014/115). All patients and controls (and their caretakers) provided written informed consent. All steps of the study conformed to the Helsinki Declaration and Good Clinical Practice guidelines.

### Sample collection

Blood samples were collected in serum separator and EDTA containing tubes. Serum and plasma from these samples were obtained via centrifugation (4000 RPM, 10 min) at Mersin University Hospital, Department of Pediatric Hematology. The samples were aliquoted, immediately frozen at − 80 °C and transferred to Hacettepe University Faculty of Medicine, Department of Medical Biochemistry, where all measurements were performed.

### Laboratory investigations

*Lipid profile* was determined by measuring total cholesterol (TC), HDL cholesterol (HDL-C), LDL cholesterol (LDL-C), VLDL-C and triglycerides (TG) in the serum by an autoanalyser (AU 680 Chemistry Analyser, Beckman Coulter, USA), using commercial kits in the clinical chemistry laboratory of Hacettepe University Hospital. Additionally, apolipoprotein A1 (Apo A1), apolipoprotein B (Apo B), free fatty acids (FFA) and lecithin cholesterol acyl transferase (LCAT) activity were measured. Commercial ELISA and EIA kits were used to measure FFA concentration (Abcam, United Kingdom) and LCAT activity (Elabscience, China), while Apo A1 and Apo B were measured via a nephelometric method in the clinical chemistry laboratory (Protein Chemistry Analyser, IMMAGE 800, Beckman Coulter, USA).

*Hemolysis* was evaluated by measuring lactate dehydrogenase (LDH), total bilirubin, direct bilirubin (AU 680 Chemistry Analyser, Beckman Coulter, USA) and haptoglobin (Hpg) in the clinical chemistry laboratory (Protein Chemistry Analyser IMMAGE 800, Beckman Coulter, USA).

*Oxidative stress* was evaluated by measuring hemin, iron and ferritin levels. Hemin was measured via enzyme immunoassay kit (Abcam, United Kingdom). Iron and ferritin were measured in the clinical chemistry laboratory (AU 680 Chemistry Analyser, Beckman Coulter, USA).

*Inflammation* was assessed by the determination of the levels of serum amyloid A (SAA), myeloperoxidase (MPO) and chitotriosidase activity. SAA and MPO were measured with commercially available ELISA kits (Abcam, United Kingdom).

Levels of alanine aminotransferase (ALT) and gamma-glutamyl transferase (GGT) as measures of hepatic function and uric acid levels as endogenous antioxidant were determined in the clinical chemistry laboratory of Hacettepe University Hospital (AU 680 Chemistry Analyzer, Beckman Coulter, USA).

### Statistical analysis

All results and data were obtained during the study were analyzed by SPSS v20 software (IBM, Armonk, NY, USA). Categorical variables were given as frequency (N) and percentage (%). Continuous variables were given as mean ± standard deviation (mean ± SD) or median (min-max) with regard to normality of distribution and statistical results. Normality of distribution was tested with the Shapiro-Wilk test. Categorical variables were compared with chi-square tests. 2-group comparisons of normally distributed continuous variables were performed with the independent samples t-test, while the Mann-Whitney U test was used to compare non-normally distributed continuous variables. > 2-group comparisons were performed with the Kruskal-Wallis test for continuous variables. The non-parametric Spearman correlation and the parametric Pearson correlation coefficients were calculated to evaluate relationships between continuous variables with regard to normality of distribution. *P* values that are lower than or equal to 0.05 were accepted as statistical significance.

## Results

We recruited 54 individuals of pediatric age group, 35 patients with SCD and 19 healthy controls. The patient group was comprised of 20 boys and 15 girls with a mean age of 13.5 ± 4.1 years, while the control group was comprised of 10 boys and 9 girls with a mean age of 13.5 ± 3.5 years. There were no differences between the groups in terms of age and sex.

There were no statistically significant differences in the clinical and laboratory findings between the patients with HbSS and HbSß^+^ genotypes. Hematological parameters of the patients and clinical history of the patients in the last year were summarized in Tables 1 and 2, respectively. When patients were compared in terms of laboratory parameters with regard to the presence or absence of complications, there were no differences in any of the laboratory parameters in any of the complications.

**Table 1:** Hematological parameters of the SCD patients

	SCD Patients (*N* = 35)
Hb (g/dL) (mean ± SD)	9.2 ± 1.1
Hb S (%)	79.8 ± 7.0
Hb F (%)	13.6 ± 7.6
Hb A_1_	1.0 ± 1.7
Hb A_2_	4.5 ± 1.7
White Blood Cell count (× 10^3^/mm^3^) (mean ± SD)	13.1 ± 4.4
Platelet count (× 10^3^/mm^3^) (mean ± SD)	464 ± 171
SCD: Sickle cell disease Hb: Hemoglobin

**Table 2:** Number of SCD patients having disease related complications in the last year

		SCD patients (N = 35)
Number of Painful crises	1–5	21
	> 5	7
Acute chest syndrome		21
Cerebrovascular event		4
Transfusion		13
Renal complication		9
Endocrine complication		6
Cardiac complication		0

SCD: sickle cell disease

Patients with SCD had hypocholesterolemia depicted by lower levels of TC (*p* < 0.001), HDL-C (p < 0.001) and LDL-C (p < 0.001), compared to controls. Apo A1 and Apo B levels were also decreased in SCD patients compared to controls. Patients with SCD were found to have significantly higher levels for TG and FFA levels than controls (Table 3).

**Table 3:** Laboratory characteristics of patients with sickle cell disease and controls (median, min-max)

	SCD Patients (N = 35)	Controls (*N* = 19)	*P* value
Age (years)	13.5 ± 4.1	13.5 ± 3.5	0.557
Gender (Boys/Girls)	20/15	10/9	0.597
TG (mg/dL)	127 (58–246)	89 (54–223)	0.042
TC (mg/dL)	116 (76–170)	167 (117–240)	0.000
HDL-C (mg/dL)	28 (20–43)	50 (32–82)	0.000
LDL-C (mg/dL)	70 (44–106)	106 (62–143)	0.000
VLDL-C (mg/dL)	25 (12–49)	18 (11–45)	0.035
Apo A1 (mg/dL)	134 (94–378)	198 (136–268)	0.000
Apo B (mg/dL)	73 (54–202)	82 (55–107)	0.016
LCAT (U/L)	133 (8–457)	285 (113–1190)	0.441
LDH (U/L)	487 (251–1122)	205 (122–293)	0.000
T. Bilirubin (mg/dL)	1.7 (0.3–4.4)	0.2 (0.1–0.6)	0.000
D. Bilirubin (mg/dL)	0.4 (0.1–0.9)	0.1 (0.0–0.1)	0.000
Hemin (mg/dL)	2.80 (0.01–22.16)	0.01 (0–0.27)	0.000
Serum Iron (μg/dL)	81 (39–237)	65 (14–143)	0.385
Ferritin (ng/mL)	112 (35–1293)	13 (6–46)	0.000
Uric acid (mg/dL)	4.7 (3.4–8.3)	4.0 (2.3–6.3)	0.025
SAA (mg/dL)	1.14 (0.27–2.63)	0.80 (0.27–1.69)	0.041
MPO (μg/L)	76 (34–189)	43 (29–56)	0.000
ALT (U/L)	21 (10–86)	12 (3–36)	0.000
GGT (U/L)	14 (8–180)	12 (3–24)	0.023

SCD: sickle cell disease, TG: triglycerides, TC: total cholesterol, HDL-C: high-density lipoprotein cholesterol, LDL-C: low density lipoprotein cholesterol, VLDL-C: very low density lipoprotein cholesterol, Apo A1: apolipoprotein A1, Apo B: apolipoprotein B, FFA: free fatty acids, LCAT: lecithin acyl cholesterol transferase, LDH: lactate dehydrogenase, SAA: serum amyloid A, MPO: myeloperoxidase, ALT: alanine transaminase, GGT: gamma-glutamyl transferase

Patients had significantly increased hemolysis reflected by increased LDH (*p* < 0.001), total bilirubin (*p* < 0.001) and direct bilirubin (p < 0.001) levels. Median Hpg concentration in the control group was 83.4 mg/dL, while Hpg concentration was undetectable in almost all patients, except three patients with levels of 6.2 mg/dL, 32.4 mg/dL and 88.2 mg/dL.

Compared to controls, hemin levels (as a measure of oxidized heme) were significantly increased among patients with SCD (*p* < 0.001). Ferritin levels were also significantly higher in the patient group (p < 0.001).

The inflammation markers, such as SAA and MPO, were significantly increased in patients with SCD. The serum levels of ALT and GGT, as markers of liver function, were significantly increased in patients compared to controls.

The correlation analyses were performed to evaluate the relationships among anemia, lipid profile, inflammation and oxidative stress. The significant correlations among these characteristics of the disease are depicted in Table 4. HDL-C is positively correlated to Hb and negatively correlated to LDL. On the other hand Apo A1 is negatively correlated to SAA. Apo B is negatively correlated to serum iron and positively correlated to ferritin.

**Table 4:** Significant correlations among lipid, hemolysis and inflammation parameters in SCD

		r	p
HDL-C	Hb	0.454	0.012
	LDH	−0.590	< 0.001
Apo A1	SAA	−0.482	0.008
Apo B	Serum Iron	− 0.360	0.050
	Ferritin	0.438	0.025
Hemin	MPO	0.383	0.040

HDL-C: high density lipoprotein cholesterol, Hb: hemoglobin, LDH: lactate dehydrogenase, Apo B: apolipoprotein B, SAA: serum amyloid A, Apo A1: apolipoprotein A1

MPO: myeloperoxidase

## Discussion

This study was conducted to understand the metabolic changes that were associated with hypocholesterolemia in the pathological course of SCD. The major findings of the study were the association between hemolysis and decreased HDL-C, as well as the link between inflammation and decreased apolipoprotein A1 levels. We suggest that, during the pathological course of SCD, the production of the HDL particle is altered, or its function is influenced.

In this study, pediatric SCD patients had hypocholesterolemia, reflected by lower total cholesterol, HDL-C and LDL-C levels. Median Apo A1 and Apo B levels in the patients were also lower than controls, suggesting either decreased production or increased catabolism of the HDL and LDL particles. The depletion of Hpg and increases in LDH and bilirubin levels demonstrate the presence of the characteristic chronic hemolysis in SCD. Increased hemin levels depicted the increased oxidative stress; whereas increased SAA and MPO demonstrated chronic inflammation. Correlation analysis showed that HDL-C levels were associated with hemolysis and anemia as well as chronic inflammation in SCD patients.

Numerous studies have shown that the levels of various lipids are altered in steady-state SCD and vaso-occlusive episodes of the disease [6, 12, 17]. Hypocholesterolemia is a hallmark of SCD and relative increases in TG levels have also been shown. TG concentration has been shown to be associated with hemolysis and inflammation [5, 12, 18, 19]. We also observed decreased TC and increased TG levels in the serum of our pediatric SCD patients, compared to healthy children (Table 1). Serum HDL-C and LDL-C levels of the patients were also lower than controls in this study.

The enzyme LCAT, which functions in reverse cholesterol transport, is carried by the HDL particle and LCAT activity is an important parameter of HDL function [20]. In a recent study, Ozturk et al. reported a reduction in LCAT protein levels in SCD [21]; however, they measured protein levels and not LCAT activity. Whereas, Soupene et al. measured LCAT specific activity and concluded that LCAT specific activity was reduced in patients with SCD, compared to controls [22]. These studies all included adult patients. In the current study, in which pediatric patients were enrolled, LCAT activities were similar in patients and controls, while Apo A1 (its cofactor) was found to be significantly lower in patients. Similar to our findings, a few studies have reported reduced Apo A1 levels at steady state and during vaso-occlusive events in patients with SCD [16, 23].

Severe chronic hemolysis was shown in our pediatric SCD patients by increased LDH levels and almost undetectable Hpg (due to scavenging toxic heme) levels. Hpg has aslo been shown to bind Apo A1 during hemolysis [24], thereby protecting Apo A1 from the detrimental effects of free oxygen radicals produced in the plasma [25]. However, severe hemolysis in SCD exhausts almost all Hpg in the plasma, causing Apo A1 to become open to the constant oxidative stress in the circulation of patients with SCD. This mechanism, the loss of defense against oxidative damage, may be a mechanism that caused the relationships between oxidative/inflammatory parameters and the levels of Apo A1 and HDL-C.

Plasma hemin levels were increased in SCD patients compared to the almost-undetectable levels in controls. Hemin is produced by the oxidation of heme [26], a molecule which was suggested to be a biomarker of disease severity in SCD [27]. It has also been shown that hemin causes oxidative damage to plasma lipoproteins, such as LDL and HDL [28]. In our study, hemin levels were found to be correlated positively with MPO levels, suggesting that hemin may play an important role in the activation of inflammation in patients with SCD, which may in turn aggravate the oxidative damage sustained by erythrocytes, endothelial cells, HDL and LDL. Taken together, these results suggest that hemin levels may contribute to inflammatory activation and possibly the oxidation of the Apo A1 of the HDL particle in SCD patients; thus causing HDL dysfunction. A recent study is linked low levels of lipoproteins to hemolysis markers, particularly to hemopexin concentrations in adults with SCD [29].

In the current study, SAA levels, as a measure of inflammation, were significantly higher among patients at steady state, which is consistent with previous studies [16, 30, 31]. There was also a negative correlation between SAA and Apo A1 levels of the SCD patients, indicating a negative relationship between chronic inflammation and HDL function. SAA is an acute phase protein synthesized by the liver during inflammation. Although vascular injury and infarction lead to increased SAA levels in SCD [32], chronic hemolysis itself is an important stimulus to increase acute phase proteins including SAA. Interestingly, SAA has been shown to have a role in splenic cholesterol transport in a mouse model mimicking hemolytic disorders [15]. Furthermore, SAA is also suggested to promote rapid cholesterol efflux in macrophages that carry phagocyted cellular debris to the spleen, a reticulo-endothelial organ [33]. Therefore, we suggest that chronic hemolysis in SCD may induce SAA production which may then contribute to hypocholesterolemia. This may also have a role in reducing reverse cholesterol transport of HDL particle. Therefore, in addition to the effects of oxidative damage, the replacement of Apo A1 by SAA seems to be associated with the dysfunction in HDL and Apo A1.

In the current study, we found MPO levels to be significantly higher among SCD patients, compared to controls. Besides, MPO levels were positively correlated with hemin levels, which can be explained by the role of MPO in both the oxidative and inflammatory processes. MPO is an enzyme that is crucial to the oxidative burst of neutrophils during the inflammatory response and it has been shown to cause oxidative damage to Apo A1 [34]. Additionally, MPO inhibition was shown to decrease vascular oxidative stress and induce vasodilation in murine models of SCD [35]. Thus, we believe that having a role in both oxidative stress and inflammation, MPO contributes to alteration of HDL particle in SCD.

According to Table 4, both HDL composition and structure seem to be affected by hemolysis and inflammation in our study. A previous study supported our view, by suggesting that Apo A1 lipoprotein composition and function are altered in SCD. The authors suggested that this was associated with the structural and functional changes seen in the HDL_2_ and HDL_3_ particles in SCD. As the level of HDL_3_ is lowered in SCD, the Apo A1 exchange rate also decreases; thus affecting cholesterol import and export by the HDL particle, which is worsened by the fact that there is an overall reduction in HDL-C levels in patients with SCD [36]. Sexias et al. previously suggested that SCD patients with low HDL and relatively increased TG might have a specific dyslipidemic phenotype of the disease [5]. However, they did not suggest any structural alteration in HDL particle as the cause of this phenotype, but their study also did not determine this with structural evidence, as the study was focused on patient characteristics (similar to ours). However, from a molecular point of view, it is very likely that the pro-inflammatory transformation of the HDL particle in SCD due to oxidative insult [37] causes lipid peroxidation (among other affects) and significantly influences all facets of lipoprotein and apolipoprotein function [14]; therefore, these alterations may be the molecular basis of the changes seen in HDL-C and Apo A1 levels and their relationships with oxidative and inflammatory parameters.

The most important limitation of this study is that, although we measured many parameters that may modify or alter the structure and function of HDL particle, we were unable to investigate the structure and composition of the HDL particle separated from SCD patients’ blood. However, we plan to investigate oxidative alterations in the HDL particle’s lipidome and proteome, particularly the oxidative post-translational modifications of Apo A1, in future studies. Besides, SCD is a rare disease and we were able to admit 35 children from a total of 70 patients under follow-up in Mersin University Hospital. The patient number is limited due to inclusion criteria limiting the age, sex and clinical condition of patients; as only steady state patients with no crisis and transfusion for the last 3 months were enrolled. Finally, although no relationships were found with regard to the presence/absence of complications within the prior year, we believe including a higher number of patients may provide a chance to assess the results of these complications in patients with SCD.

## Conclusion

Pediatric SCD patients in this study had hypocholesterolemia reflected by low TC, LDL-C and HDL-C levels. We suggest that the cumulative effect of chronic hemolysis, oxidative stress and inflammation in SCD may result with a pathological structural alteration in HDL. Several explanations about these relationships were reported in this study under the light of prior research on this topic. Briefly put, the slowing down of reverse cholesterol transport (induced by increased SAA) in response to chronic inflammation and/or oxidative stress may be an important cause of hypocholesterolemia in SCD.

## Data Availability

Not applicable.

## Abbreviations

Apo B: Apolipoprotein B
ApoA1: Apolipoprotein A1
FFA: Free fatty acids
Hb: Hemoglobin
HbS: Hemoglobin sickle
HbSS: Homozygous SCD
HbSß^+^: Hemoglobin sickle beta thalassemia
HDL: High density lipoprotein
HDL-C: High density lipoprotein cholesterol
Hpg: Haptoglobin
LCAT: Lecithin cholesterol acyl transferase
LDH: Lactate dehydrogenase
LDL: Low density lipoprotein
LDL-C: LDL cholesterol
MPO: Myeloperoxidase
SAA: Serum amyloid A
SCD: Sickle cell disease
TC: Total cholesterol
TG: Triglycerides
VLDL: Very low density lipoprotein
VLDL-C: Very low density lipoprotein cholesterol
